# Risk of Short-Term Stroke in Non-High-Risk Patients Undergoing Transcatheter Aortic Valve Replacement vs Surgical Aortic Valve Replacement: A Systematic Review and Meta-Analysis

**DOI:** 10.31083/RCM49672

**Published:** 2026-09-30

**Authors:** Haochen Liu, Zhidan Xiao, Jiahao Zou, Xiaoping Peng

**Affiliations:** ^1^Department of Cardiology, The First Affiliated Hospital, Jiangxi Medical College, Nanchang University, 330031 Nanchang, Jiangxi, China; ^2^Jiangxi Hypertension Research Institute, 330006 Nanchang, Jiangxi, China; ^3^Jiangxi Medical College, Nanchang University, 330031 Nanchang, Jiangxi, China

**Keywords:** aortic valve stenosis, transcatheter aortic valve replacement, heart valve prosthesis implantation, stroke, meta-analysis

## Abstract

**Background:**

To compare the incidence of stroke at 1 and 2 years following transcatheter aortic valve replacement (TAVR) versus surgical aortic valve replacement (SAVR), aiming to inform clinical decision-making regarding the safety and effectiveness of these interventions.

**Methods:**

A random-effects meta-analysis was conducted including 9 randomized controlled trials (RCTs) with a total of 9865 patients. The primary endpoint was the incidence of stroke at 1 and 2 years post-procedure.

**Results:**

At 1 year, TAVR was associated with a lower incidence of stroke compared with SAVR (odds ratio (OR), 0.77; 95% confidence interval (CI), 0.61–0.98; *p* = 0.03). No significant difference was observed at 2 years (OR, 0.87; 95% CI, 0.65–1.17; *p* = 0.37).

**Conclusions:**

TAVR significantly reduces the risk of stroke within 1 year post-procedure compared with SAVR; however, this effect was not maintained at 2 years. These findings highlight the early benefit of TAVR, while the long-term advantage requires further investigation.

**The PROSPERO Registration:**

This study has been registered with PROSPERO, registration number: CRD42025636191, https://www.crd.york.ac.uk/PROSPERO/view/CRD42025636191.

## 1. Introduction

The lifetime risk of stroke globally has increased from 22.8% in 1990 to 24.9% in 2016 [1]. In 2019, stroke continued to be the third leading cause of death and disability worldwide. From 1990 to 2019, its burden, as measured by absolute case numbers, saw a significant increase [2]. Stroke survivors frequently encounter significant cognitive and mobility impairments [3], with approximately one-third experiencing heightened health anxiety, increased rates of depression, and a diminished quality of life [4]. The treatment of severe aortic stenosis involves notable differences between transcatheter aortic valve replacement (TAVR) and surgical aortic valve replacement (SAVR), especially concerning the risk of early stroke and transient ischemic attacks [5].

Research indicates that TAVR may more effectively improve patients’ health status in the long term and result in better postoperative outcomes [6,7]. A study by Zorn GL 3rd et al. [8] found that among patients with high surgical risk and severe aortic stenosis, the incidence of severe prosthesis-patient mismatch (PPM) was more prevalent in those undergoing SAVR compared to those treated with TAVR.

The study conducted by Fan P et al. [9] demonstrates that the all-cause mortality rate and the incidence of new-onset atrial fibrillation following TAVR are significantly lower than those observed after SAVR. This finding reinforces the recommendation of TAVR as the preferred treatment for patients with aortic stenosis [9].

However, some researchers argue that there are no significant differences between the two procedures in terms of health status improvement [10,11]. Regarding complications, several studies have shown that both methods do not exhibit statistically significant differences in all-cause mortality, myocardial infarction, or major infections [12].

Conversely, others contend that the early risks associated with TAVR are greater than those linked to SAVR [13]. In patients with a smaller aortic valve area index, the TAVR group displays a higher risk compared to the SAVR group, alongside a greater incidence of neurological events [14].

Currently, there is limited comprehensive research examining both the short-term and long-term stroke risk in non-high-risk patients undergoing TAVR or SAVR. While some studies have explored stroke incidence across various risk groups, there is still a lack of detailed insight into the early stroke risk specifically for this patient population. Therefore, it is important to investigate the relative risk of early stroke in non-high-risk patients undergoing either TAVR or SAVR to support clinical decision-making and enhance patient outcomes.

This study will perform a systematic review and meta-analysis to compare the stroke risk in non-high-risk patients who undergo TAVR with those who undergo SAVR at one and two years post-treatment. Our objective is to enhance the understanding of the safety and effectiveness of these two procedures.

## 2. Methods

This systematic review and meta-analysis were conducted in accordance with the Preferred Reporting Items for Systematic Reviews and Meta-Analyses (PRISMA) guidelines. The study sources included two primary databases: PubMed and Embase. The search encompassed studies published between 2013 and November 1, 2024, and was restricted to articles in English. We specifically sought trials that compared TAVR and SAVR and reported stroke outcomes, utilizing keywords such as “TAVR”, “SAVR”, “stroke”, and their relevant synonyms. The PRISMA checklist is provided in the **Supplementary Material**.

## 3. Study Selection

Following a comprehensive search across the databases, the identified studies were imported into the EndNote reference library for organization, with duplicates systematically removed. Two authors, HCL and ZDX, independently reviewed the titles and abstracts based on predefined eligibility criteria, excluding studies that did not meet the established standards. After this initial screening, the full texts of potentially relevant studies were retrieved for thorough evaluation to ensure compliance with the defined criteria.

### 3.1 Inclusion Criteria

Study Design: This systematic review will include only randomized controlled trials (RCTs) that report and compare the incidence of stroke between TAVR and SAVR. A minimum follow-up period of one year is required.

Participants: Eligible participants will consist of patients aged 18 years and older who have been diagnosed with aortic stenosis and who have undergone either TAVR or SAVR. Our study targets non-high-risk patients, specifically those categorized as low-risk and moderate-risk. Low-risk patients are defined as those with a Society of Thoracic Surgeons Predicted Risk (STS) score of less than 4% [15], while moderate-risk patients are those with an STS score greater than 4% but less than 8%. High-risk patients are defined as those with an STS score exceeding 8%. This classification is consistent with widely accepted clinical practices.

Language: Only studies published in English will be included. During study selection and data extraction, we verified the risk profile of each included study based on the information reported in the original articles. Seven of the nine included trials explicitly defined their populations as low- or intermediate-risk according to STS criteria, whereas the remaining two did not report specific STS values; for these studies, eligibility was judged based on the baseline characteristics and overall risk descriptions provided in the original reports.

### 3.2 Exclusion Criteria

Study Design: Non-randomized controlled trials will be excluded from this review, as will studies that do not include patients who underwent TAVR or SAVR.

Participants: Patients under the age of 18, those who did not undergo TAVR or SAVR, and individuals who have had surgeries other than TAVR or SAVR will be excluded.

Language: Studies published in languages other than English will be excluded.

The inclusion and exclusion criteria for this study have been established to ensure that only high-quality randomized controlled trials relevant to the research question and with a low risk of bias are considered. This rigorous selection process aims to provide reliable evidence for comparing stroke rates between TAVR and SAVR, thereby enhancing the validity of the analysis and the robustness of the research findings.

## 4. Data Extraction

In the data extraction phase, relevant information from selected studies was compiled into an Excel spreadsheet. Each piece of data, including study characteristics, participant demographics, and key outcomes, was recorded to ensure clarity and accessibility for further statistical analysis. The use of Excel also allowed for efficient sorting and retrieval of data essential for comprehensive analysis. The 1-year and 2-year time points were selected as clinically meaningful intervals for evaluating early and intermediate stroke risk after TAVR and SAVR. Most included randomized controlled trials reported stroke outcomes at these intervals, enabling robust and comparable meta-analysis. While the original trials varied in pre-specified endpoints, several studies explicitly designated 1-year or 2-year stroke incidence as primary or secondary outcomes. For trials without pre-specified stroke endpoints at these exact times, the longest available follow-up data closest to 1 and 2 years were used, consistent with standard meta-analytic practice to maximize data utilization.

## 5. Methodological Quality

The methodological quality of each included study was assessed using the Cochrane Collaboration’s risk of bias tool for randomized controlled trials. This evaluation focused on various aspects of study design, including internal validity and potential sources of bias. Each study was critically reviewed to determine its overall quality, and a risk of bias rating was assigned. The results were visually summarized using a color-coded system that categorized studies as low, high, or uncertain risk of bias, which facilitated clearer interpretation and identification of limitations within the evidence. This comprehensive evaluation was essential for drawing valid conclusions and making informed recommendations (Fig. 1 and Fig. 2).

**Fig. 1.**
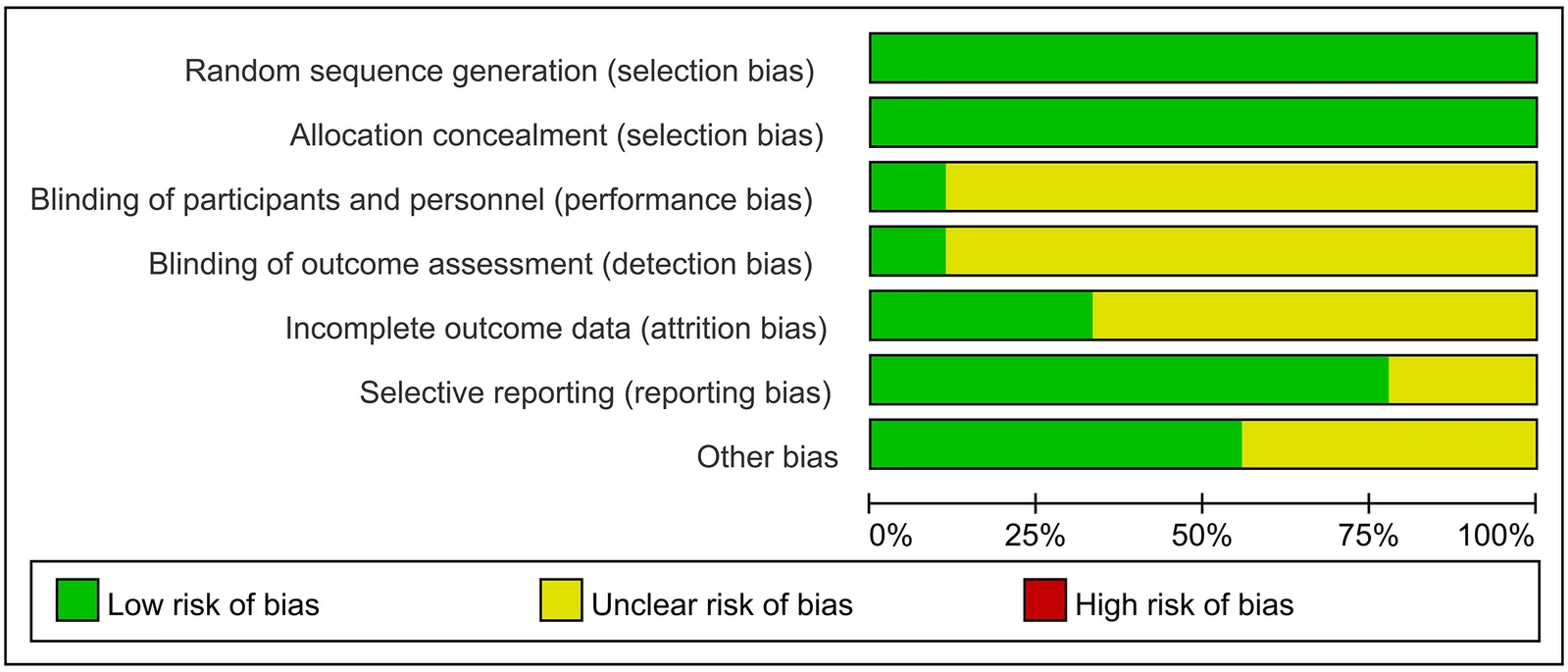
**Risk of bias graph**.

**Fig. 2.**
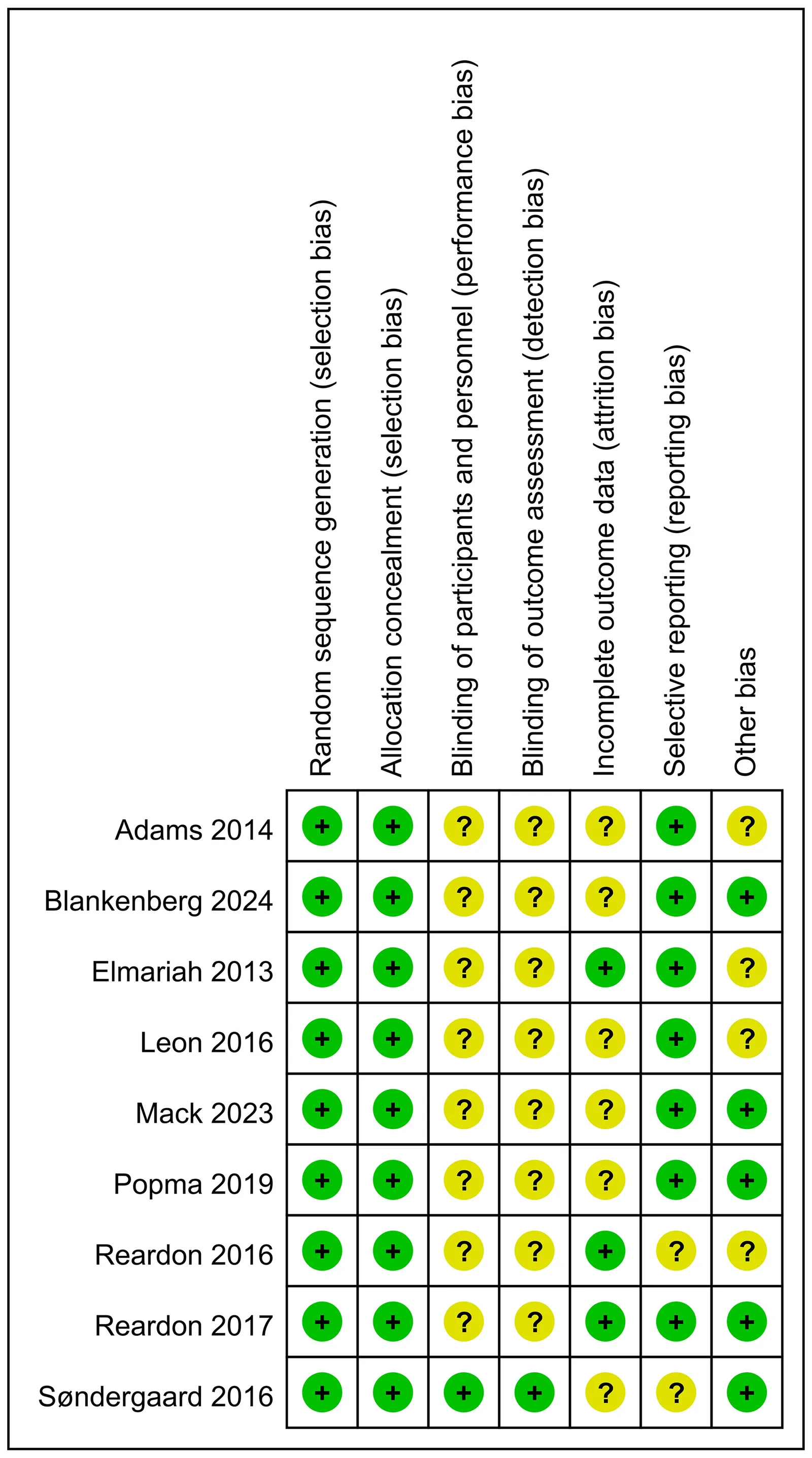
** Risk of bias summary**.

## 6. Strategy for Data Synthesis

All statistical analyses were performed using Review Manager (RevMan, version 5.3; The Cochrane Collaboration, London, UK). For dichotomous outcomes, pooled effect estimates were calculated as odds ratios (ORs) with 95% confidence intervals (CIs). Heterogeneity among studies was assessed using Cochran’s Q test and the I^2^ statistic. An I^2^ value of 0%–25% was considered low heterogeneity, 25%–50% moderate heterogeneity, 50%–75% substantial heterogeneity, and >75% considerable heterogeneity. A *p* value < 0.10 in the Q test was considered indicative of statistically significant heterogeneity. Given the anticipated clinical and methodological variability across the included trials (e.g., differences in patient risk profiles, valve types, and follow-up durations), a random-effects model (DerSimonian–Laird method) was used as the primary analytical approach. Fixed-effect models were additionally explored in sensitivity analyses when appropriate. Forest plots were generated to visually display individual and pooled effect sizes. Publication bias was assessed using funnel plots by examining the symmetry of effect estimates against their standard errors. To assess potential publication bias, a funnel plot was initially constructed. Considering that the number of included studies was less than 10, which may limit the interpretability of the funnel plot, we further conducted Egger’s regression test. A *p*-value < 0.05 was considered indicative of significant publication bias.

## 7. Results

After a comprehensive search of the selected databases, a total of 303 articles were identified, including 143 from PubMed and 160 from Embase. Following the application of exclusion criteria, 9 articles were ultimately included for evaluation and analysis. The search strategy and results are summarized in Fig. 3.

**Fig. 3.**
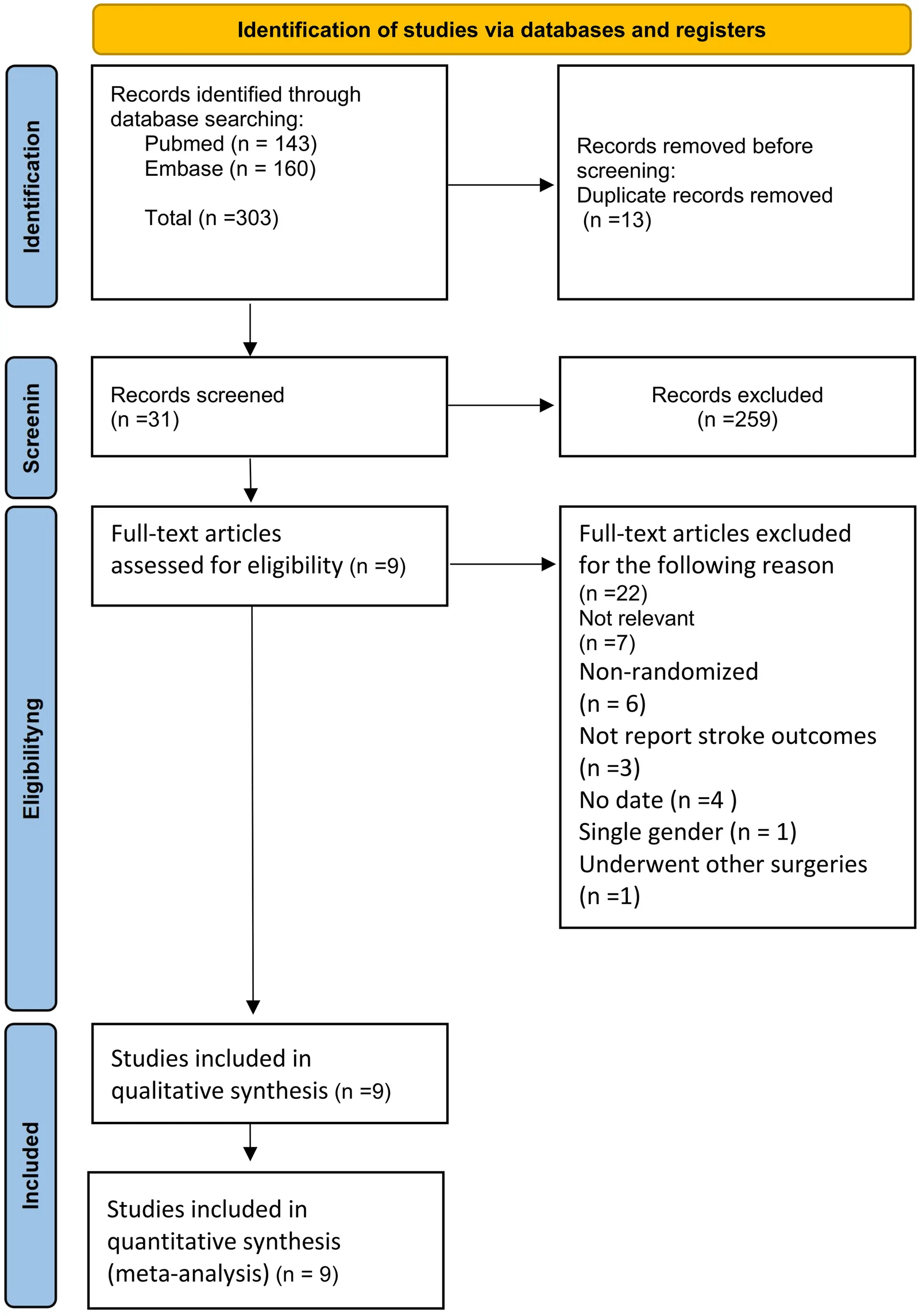
**Flow diagram from Preferred Reporting Items for Systematic Reviews and Meta-Analyses (PRISMA) 2020**.

In total, 9865 patients were included in the analysis, with 5051 undergoing TAVR and 4814 undergoing SAVR after randomization. Among the studies, two trials reported a follow-up duration of 1 year, four trials lasted for 2 years, and three trials had a duration of 5 years. The weighted mean follow-up duration across all trials was 2.38 years. Basic information from each included study is presented in Table 1 (Ref. [16,17,18,19,20,21,22,23,24]).

**Table 1.** **Basic demographics of studies**.

Author	Study type	Duration of study	Total population (n)	Experimental population (n)	Control (n)	Age, mean ± SD (years)	Follow up	Stroke definition
Adams et al., 2014 [16]	RCT	1 y	747	390	357	83.1 ± 7.1	1 y	Any stroke
Blankenberg S et al., 2024 [17]	RCT	5 y	1414	701	713	74.3 ± 4.6	1 y	Any stroke
Elmariah S et al., 2013 [18]	RCT	1 y	636	332	304	83.7 ± 7.0	2 y	Any stroke
Leon MB et al., 2016 [19]	RCT	3 y	2032	1011	1021	81.5 ± 6.7	2 y	Any stroke
Mack MJ et al., 2023 [20]	RCT	2 y	950	496	454	73.3 ± 5.8	5 y	Any stroke
Popma JJ et al., 2019 [21]	RCT	3 y	1403	725	678	74.4 ± 5.8	2 y	Any stroke
Reardon MJ et al., 2017 [22]	RCT	2 y	1657	863	794	79.8 ± 6.2	2 y	Any stroke
Søndergaard L et al., 2016 [23]	RCT	4 y	276	142	134	79.1 ± 4.8	5 y	Any stroke
Reardon MJ et al., 2016 [24]	RCT	3 y	750	391	359	83.2 ± 6.7	5 y	Any stroke

RCT, randomized controlled trial.

A meta-analysis of the 9 relevant studies was conducted to evaluate stroke rates at both 1 year and 2 years as outcome metrics. The results revealed that 9 studies reported the 1-year stroke rate, while 6 studies provided data on the 2-year stroke rate.

For the 1-year stroke outcome, moderate heterogeneity was observed (I^2^ = 32.0%; Cochran’s Q test *p* = 0.16). Accordingly, a random-effects model was applied, indicating that the degree of clinical heterogeneity was acceptable. A total of 9865 patients were included in the pooled analysis. The combined effect estimate showed an OR of 0.82 (95% CI, 0.69–0.97), suggesting that the true effect size is likely to lie within this interval. The Z statistic was 2.12 (*p* = 0.02), indicating statistical significance. In the forest plot, the pooled effect (diamond) was located to the left of the line of no effect and did not cross it. Based on data from nine trials, patients undergoing TAVR had a lower incidence of stroke at 1 year compared with those undergoing conventional SAVR, suggesting a potential advantage of TAVR in reducing early stroke risk (Fig. 4).

**Fig. 4.**
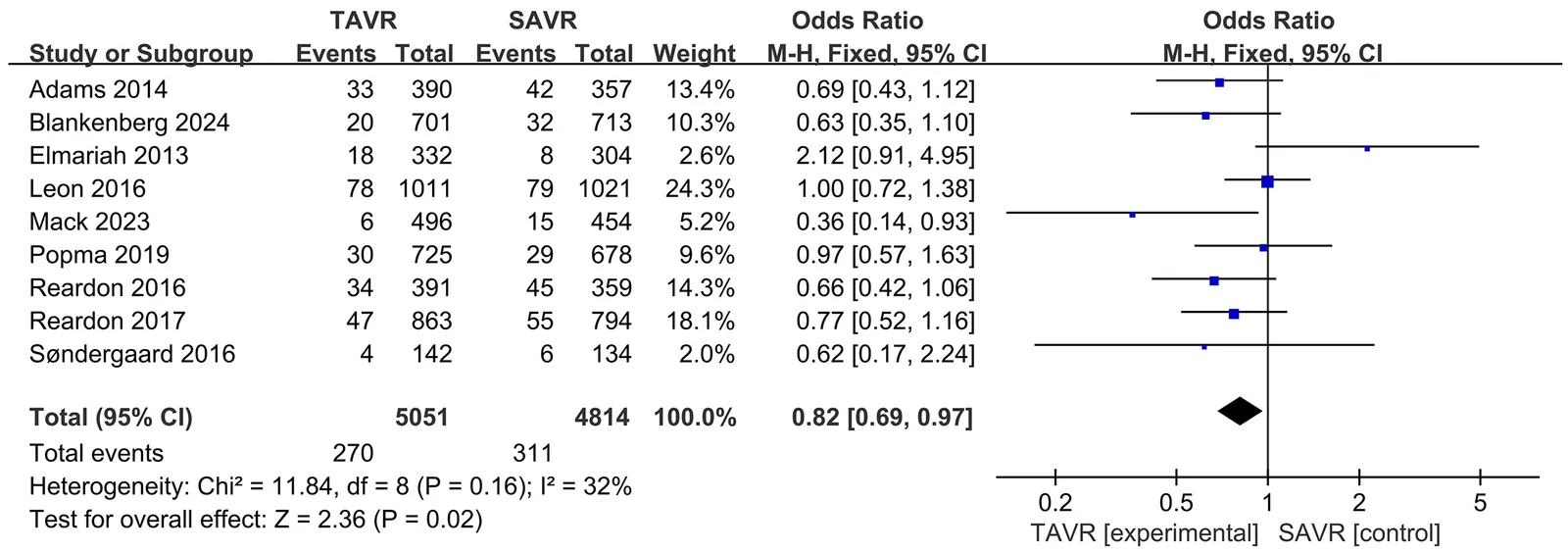
**Forest plot of 1-year stroke incidence in transcatheter aortic valve replacement (TAVR) vs surgical aortic valve replacement (SAVR) procedures**.

For the 2-year stroke outcome, moderate heterogeneity persisted (I^2^ = 46%; Cochran’s Q test *p* = 0.10), and a random-effects model was likewise employed. The pooled sample comprised 6301 patients. The combined effect estimate was OR = 0.87 (95% CI, 0.65–1.17), indicating that the true effect size is likely to fall within this range. The Z statistic was 0.91 (*p* = 0.37), showing no statistically significant difference. In the forest plot, the pooled effect (diamond) crossed the line of no effect. Analysis of six trials demonstrated no significant difference in the incidence of stroke at 2 years between TAVR and SAVR. Individual study estimates and their corresponding weights are presented in the forest plot (Fig. 5).

**Fig. 5.**
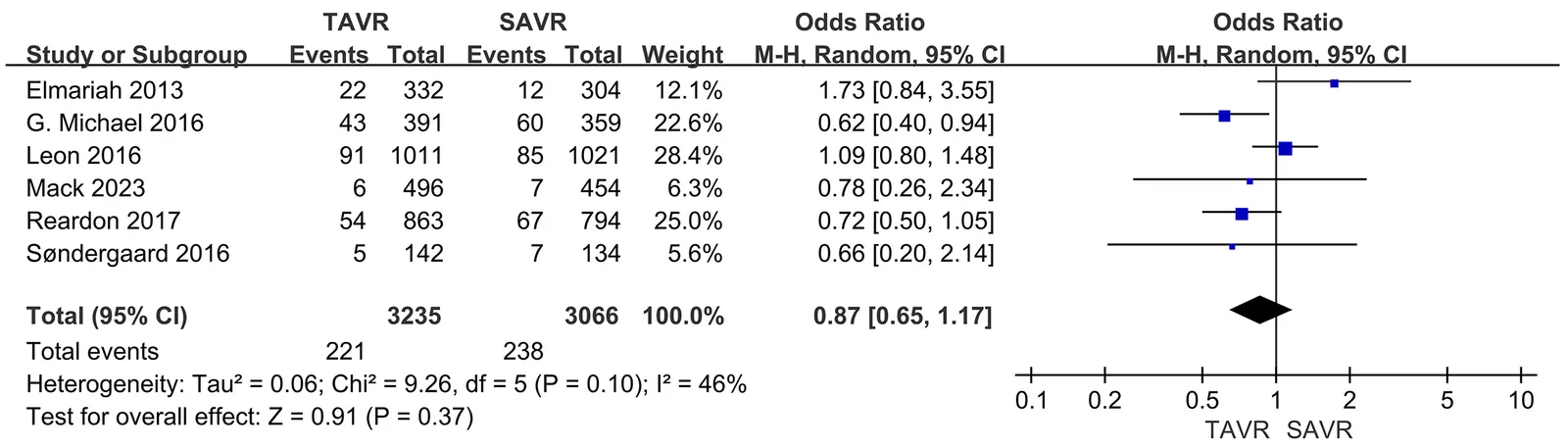
**Forest plot of 2-year stroke incidence in TAVR vs SAVR procedures**.

## 8. Publication Bias

To further assess the risk of bias, we generated a funnel plot for the studies included in this meta-analysis, thereby enhancing the robustness of the data results. The distribution of studies exhibited symmetry, with most studies concentrated at the top of the funnel and evenly distributed across all quadrants, indicating that the influence of smaller sample studies was minimal. This pattern suggests a low risk of publication bias, reinforcing the reliability of the results (Fig. 6). Consistently, the Egger’s regression test yielded a *p*-value of 0.60, indicating no significant publication bias among the included studies.

**Fig 6.**
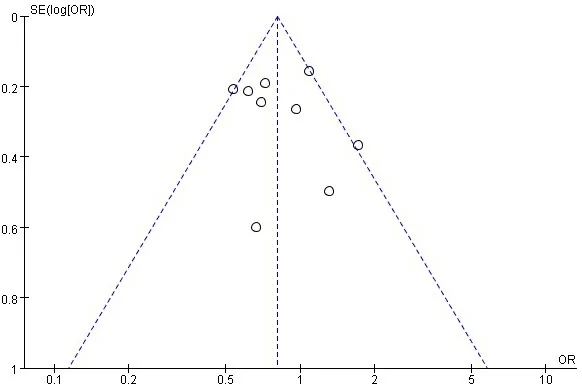
**Funnel plot assessing publication bias in studies of stroke incidence following TAVR vs SAVR procedures**.

## 9. Discussion

Notably, perioperative stroke is more commonly associated with procedural factors, including device manipulation, embolization of calcific or atherosclerotic debris, and transient hemodynamic instability [25]. In contrast, long-term stroke is more likely influenced by patients’ baseline characteristics and ongoing thromboembolic risk, such as atrial fibrillation, systemic atherosclerotic burden, and prior cerebrovascular disease [26]. This study primarily focuses on stroke events occurring during the postoperative follow-up period.

In this meta-analysis, we compared early stroke rates between TAVR and SAVR in patients with aortic stenosis. The study included a total of 9865 patients who participated in 9 studies, with patients randomly assigned to either the experimental group or the control group. The experimental group comprised 5051 patients who underwent TAVR, while the control group included 4814 patients who received SAVR.

To facilitate a comprehensive comparison, we categorized clinical events based on stroke rates at 1 year and 2 years post-intervention. This approach provides practical evidence for discussing the relevant outcomes of TAVR and SAVR with patients and healthcare professionals over different clinical time frames. The results of this study indicate that patients undergoing TAVR experience a significantly lower incidence of stroke within one year post-surgery compared to those undergoing SAVR. This finding has substantial clinical implications. Given the profound impact of stroke on patients’ quality of life and overall prognosis, TAVR may present a distinct advantage in reducing early stroke risk, positioning it as a preferred treatment option for patients with aortic stenosis, particularly in circumstances necessitating rapid health improvement.

The moderate heterogeneity observed in the 1-year and 2-year analyses (I^2^ ≈ 45%–46%) may be attributed to several factors. First, variations in patient risk stratification (low vs. intermediate risk) across trials could influence stroke outcomes. Second, differences in valve platforms and procedural techniques, particularly across different generations of TAVR devices, may have contributed to variability. Third, inconsistencies in follow-up duration definitions and outcome assessment methods among studies may also have introduced heterogeneity. Despite these differences, the direction of the pooled effect remained consistent, supporting the robustness of our findings. The definition of non-high-risk patients was not entirely consistent across the included trials. Although 7 of the 9 included studies explicitly defined their populations as low- or intermediate-risk according to STS criteria, 2 studies did not report specific STS values. Because individual-level data were unavailable, we were unable to reclassify all participants using a uniform STS threshold. Therefore, this issue may represent a potential source of clinical heterogeneity and may limit the generalizability of our findings.

The study conducted by Mack et al. [20] demonstrated a significant difference in stroke incidence within one year between the TAVR and SAVR groups, reporting rates of 1.2% and 3.3%, respectively, with a hazard ratio (HR) of 0.36 (95% CI: 0.14–0.92; *p* < 0.05). Similarly, research by Takagi et al. [27] found that transcatheter aortic valve implantation (TAVI) could reduce all-cause mortality at both 30 days and one year compared to SAVR. Furthermore, Gargiulo et al. [28] reported that TAVI may yield comparable or even superior early and mid-term outcomes relative to SAVR in adults with aortic stenosis, including those at low to intermediate risk. These findings further corroborate our results, suggesting that patients with aortic stenosis undergoing TAVR have a lower incidence of stroke compared to those receiving conventional surgical interventions.

Additionally, a nationwide observational analysis by Ismayl et al. [29] found that TAVR is increasingly performed in patients with aortic stenosis and coronary artery disease (AS-CS), and is associated with a lower incidence of in-hospital complications compared to SAVR.

In contrast, when comparing 2-year stroke rates between TAVR and SAVR, this study did not reveal significant differences. The research conducted by Chen et al. [30] indicated that, among intermediate-risk patients with severe aortic stenosis undergoing either procedure, the relative risks of adverse clinical outcomes at 2 years were similar for both groups. Nonetheless, the reduced early stroke risk remains a critical advantage of TAVR.

Importantly, while TAVR demonstrated a significantly lower incidence of stroke within the first year compared to SAVR, no significant difference was observed at the 2-year follow-up. This finding is consistent with prior studies [31] indicating that perioperative strokes are predominantly influenced by procedural factors (e.g., device manipulation, embolization, transient hemodynamic instability), whereas long-term stroke risk is largely determined by patients’ baseline characteristics such as atrial fibrillation, systemic atherosclerosis, and prior cerebrovascular disease.

Even if the early reduction in stroke risk does not appear to confer a statistically significant long-term advantage, it remains clinically relevant, given the substantial impact of early stroke on patients’ quality of life and overall prognosis. Therefore, the early benefit of TAVR should be considered meaningful in clinical decision-making, while its translation into sustained long-term advantage requires further investigation through extended follow-up studies.

Conversely, Miller et al. [13] found that neurologic events were more prevalent in patients undergoing TAVR, with an incidence rate twice that of the SAVR group (8.3% vs. 4.3% at 1 year; *p* = 0.04). However, they observed no significant difference in the rate of major strokes between the two groups (5.1% vs. 2.4% at 1 year; *p* = 0.07) [13]. This discrepancy raises important questions regarding potential confounding factors that might influence stroke incidence in these patients.

The study by Panchal et al. [32] revealed that while 23 individual studies highlighted specific advantages for each technique within distinct cohorts, a combined analysis of over 65,000 data points through stratified surgical evaluation showed that TAVR is comparable to SAVR in low-risk and intermediate-risk populations, and only superior in high-risk groups for short- and mid-term outcomes.

Diegoli et al. [33] indicated that, compared to SAVR, TAVR can reduce the risks of atrial fibrillation (AF) and life-threatening bleeding in patients at high surgical risk. In those with intermediate surgical risk, TAVR was associated with lower risks of AF, life-threatening bleeding, and acute renal failure. For patients with low surgical risk, TAVR demonstrated reduced risks of death, stroke, AF, life-threatening bleeding, and acute renal failure; however, a higher risk of permanent pacemaker implantation was noted [33]. Thus, risk stratification may help elucidate these findings.

Llerena-Velastegui et al. [34] conducted a meta-analysis in 2024 comparing the outcomes of TAVR and SAVR over a period of 1 to 5 years. The study indicated that TAVR is not inferior to SAVR in reducing long-term stroke incidence [34]. However, it also noted that TAVR does not significantly diminish the risk of stroke in patients with heart disease compared to traditional surgery over the long term [34]. Consequently, healthcare professionals should remain vigilant regarding this early complication to provide effective patient management.

Our study distinguishes itself from Llerena-Velastegui’s by its specific focus on data collected from 1 to 2 years post-procedure, rather than the broader 1 to 5-year timeframe. This targeted approach aims to clarify the early effects of these surgical interventions on patient outcomes and allows our research to more effectively control for biases stemming from confounding factors associated with extended observation periods.

Regarding age-related risk, although the included studies did not allow for a standardized determination of the precise peak age for stroke occurrence, current guidelines recommend SAVR as the preferred treatment for patients younger than 70 years [35]. However, the use of TAVI in this age group has been increasing rapidly, with a growing proportion of such patients represented in overall TAVI cohorts [36,37]. Conversely, some studies indicate that, compared with younger individuals, older patients (>82 years), despite having significantly lower body mass index and smaller aortic valve area, do not exhibit significant differences in device implantation success, all-cause mortality, stroke incidence, or access-site complications between the two age groups [38].

In addition, certain patient phenotypes may be particularly vulnerable to stroke after valve replacement, including older individuals, patients with previous stroke/transient ischemic attack (TIA), atrial fibrillation, severe valvular or aortic calcification, peripheral vascular disease, chronic kidney disease, and frailty. However, these observations should be interpreted with caution because phenotype-specific data were inconsistently reported across studies.

In summary, this study underscores the potential advantages of TAVR in the treatment of aortic stenosis by analyzing stroke risk at 1 and 2 years post-procedure. It is recommended that healthcare professionals prioritize this surgical approach in clinical practice to reduce the early risk of stroke in patients.

## 10. Limitations

Our study presents several limitations. First, only nine relevant studies were included, and the adequacy of the sample size was not evaluated, potentially impacting the reliability of our conclusions regarding early stroke incidence in patients undergoing TAVR or SAVR. Second, the inclusion of relevant studies was restricted by language and publication dates, which may have introduced bias into our analysis. Third, the definition of non-high-risk patients was not entirely consistent across the included trials. Although 7 of the 9 included studies explicitly defined their populations as low- or intermediate-risk according to STS criteria, 2 studies did not report specific STS values. Because individual-level data were unavailable, we were unable to reclassify all participants using a uniform STS threshold. Therefore, this issue may represent a potential source of clinical heterogeneity and may limit the generalizability of our findings.

## 11. Conclusion

Based on our research findings, TAVR may emerge as the preferred surgical approach for reducing early stroke risk in non-high-risk patients. Our study demonstrates that TAVR significantly reduces the incidence of stroke within one year following the procedure, providing crucial evidence for clinicians in their selection of surgical methods. Considering the profound impact of stroke on patients’ quality of life and prognosis, the early advantages of TAVR highlight its considerable potential in managing patients with aortic stenosis. Therefore, healthcare professionals should prioritize TAVR in clinical practice as a strategy to enhance patient health and mitigate the risk of early stroke.

## Data Availability

The data supporting the findings of this study are available from the published studies included in this review. Additional data generated or analyzed during the current study are available from the corresponding author upon reasonable request.
